# Interface Characteristics of Tungsten-Particle-Reinforced Zr-Based Bulk-Metallic-Glass Composites with Different Tungsten Particle Sizes

**DOI:** 10.3390/ma16155212

**Published:** 2023-07-25

**Authors:** Haoyu Jin, Huie Hu, Junhan Chi, Yunfei Ma, Xiaohong Su

**Affiliations:** 1 Department of Chemistry and Materials, Naval University of Engineering, Wuhan 430033, China; jianbukecui549549@163.com (H.J.); cjh77881221@163.com (J.C.); surrey0908@sina.com (X.S.); 2 State Key Laboratory of Materials Processing and Die & Mould Technology, School of Materials Science and Engineering, Huazhong University of Science and Technology, Wuhan 430074, China; mayunfei@hust.edu.cn

**Keywords:** bulk-metallic-glass composites, tungsten particles, interface characteristics, nanoindentation

## Abstract

This study investigated the interfacial characteristics of tungsten-particle-reinforced Zr-based bulk-metallic-glass composites (Wp/Zr-BMGs) with varying tungsten-particle sizes. To this end, Wp/Zr-BMGs with three different Wp sizes were fabricated using spark plasma sintering. Subsequently, the microstructures and interfacial structures of the Wp/Zr-BMGs were extensively examined, and the mechanical properties of the microzone at the Wp/Zr-BMG interface were evaluated using a nanoindentation method. The results revealed that the interfaces of Wp/Zr-BMGs, irrespective of the Wp size, exhibited dissolution-diffusion characteristics. Moreover, the thickness of the interface diffusion layer was positively correlated to the size of Wp. The addition of Wp enhanced the elastic modulus and hardness of Zr-BMGs at the interface, as these effects are inversely related to the Wp size. Furthermore, this study established a relationship between the interfacial mechanical properties and the interfacial characteristics of particle-reinforced bulk-metallic-glass composites. Thus, this study can serve as a guide for future research in the field of Wp/Zr-BMGs and similar particle-reinforced composites.

## 1. Introduction

Bulk metallic glass (BMG) is a solid alloy characterized by long-range disorder and short-range order, formed through rapid cooling of the molten alloy. BMGs exhibit exceptional mechanical, physical, and chemical properties due to the absence of common defects found in crystalline alloys, such as dislocations, crystal boundaries, and segregation [1,2,3,4,5,6]. Zr-based bulk metallic glass (Zr-BMG) possesses remarkable glass-forming ability and excellent attributes such as high strength, hardness, wear resistance, and corrosion resistance, thereby garnering significant attention. However, its brittleness and lack of macroscopic plasticity greatly limit its practical applications [7,8,9,10]. To enhance the overall performance of Zr-BMG and meet practical requirements, the addition of a second phase to form bulk metallic glass composites with improved plasticity and toughness has been explored. This approach effectively prevents the expansion of shear bands during fracture, reducing the brittleness of the BMG [11,12].

Tungsten has emerged as an ideal second-phase material due to its high density, high melting point, good thermal conductivity, stability, and excellent wettability with Zr-BMG [13,14]. Tungsten-reinforced Zr-based bulk-metallic-glass composites (W/Zr-BMGs) offer enhanced density and plasticity while retaining the high strength and hardness of Zr-BMGs. W/Zr-BMGs are considered promising armor-piercing core materials that could potentially replace tungsten alloys, given their penetration “self-sharpness” akin to depleted uranium alloys. Consequently, they have become a focal point of research for domestic and international scholars [15,16,17,18].

The internal structure of Wp/Zr-BMGs comprises a Zr-BMG matrix phase, a reinforcement phase, and an interface between them. The interface serves as the bonding region between the matrix and reinforcement phases, exerting a crucial influence on load transmission, strengthening mechanisms, deformation, and fracture processes within the material [19,20,21,22]. Consequently, investigating the interface becomes imperative when evaluating composite material performance.

Kou et al. [23] found that diffusion bonding joints between Zr_41.25_Ti_13.75_Cu_12.5_Ni_10_Be_22.5_ BMG and copper can be formed at super-cooled liquid region temperatures, and the crystalline phases transformed from the amorphous state will accelerate the atomic diffusion at the interface. Georgarakis et al. [24] reviewed the factors affecting the thickness of the interface layer in metallic glass-reinforced metal matrix composites, such as processing conditions and the interaction between the two phases, and found that the reactivity and diffusivity of several elements influenced the resultant interfacial structure. Moreover, previous studies have explored the interfacial characteristics of tungsten fiber (Wf)-reinforced Zr-based bulk-metallic-glass composites (Wf/Zr-BMGs) prepared via infiltration casting. Mofid [25] conducted research on Wf/Zr-BMGs with different infiltration times, observing pronounced interfacial reactions and the formation of a Zr/W-rich reaction layer when the infiltration time was 15 min. Moreover, Li et al. [13] investigated the interfacial reaction and bonding mechanism of Wf/Zr-BMGs prepared via the infiltration casting water quenching method, revealing a bonding mechanism that involved mutual diffusion of components and a direct peritectic reaction between Zr in the matrix and Wf, leading to the formation of W_5_Zr_3_. Furthermore, Feng [26] examined the interfacial microstructure of Wp/Zr-BMGs prepared using the infiltration casting water quenching method, observing bidirectional diffusion of elements at the interface along with the generation of numerous interfacial reaction products, particularly in samples with larger Wp diameters.

Although recent research has focused on the interface bonding mechanism of W/Zr-BMGs, there remains a dearth of studies on the micromechanical properties of the composite interface microzone. Nevertheless, limited research has been conducted on the influence of Wp size on the interface characteristics of Wp/Zr-BMGs. Hence, this study focused on Wp/Zr-BMGs with varying Wp particle sizes, which were fabricated using spark plasma sintering (SPS). It explored the interface bonding mechanism of composites with different Wp sizes and examined the impact of Wp size on the mechanical properties of the interface microzone. The study aimed to initially establish a relationship between the interface mechanical properties and the interface characteristics of particle-reinforced bulk-metallic-glass composites.

## 2. Experiment

Wp/Zr-BMGs with three sizes of Wp were sintered through SPS (sintering temperature 678 K, sintering pressure 500 MPa [10]); the three average sizes of Wp are 30, 75, and 250 μm. The volume fraction of Wp was maintained at 50%. The raw material of metallic glass matrix is powder whose size is from 0 μm to 53 μm. The sample size was Φ10 mm × 15 mm, and the nominal chemical composition of Zr-BMG was Zr_55_Cu_30_Al_10_Ni_5_ (at.%). X-ray diffractometry (XRD; Bruker D8 Advance) was used to analyze the phases of the composites with different Wp sizes. The X-ray tube voltage was 40 kV, the tube current was 40 mA, the scanning angle range was 10° to 90°, the characteristic radiation was Cu-Kα, the step size was 0.02°, and the scanning speed was 10°/min.

Based on field-emission scanning electron microscopy (FE-SEM; ZEISS GeminiSEM 300, Jena, Germany) and energy dispersive spectrometry (EDS; Smartedx, UK), the overall distribution of Wp in the composites with different Wp sizes, interface bonding, and contents of each component element at the interface were observed. The accelerating voltage was 15 kV, and the magnification ranged from 100× to 10 k×.

A Bruker Hysitron TI980 nanoindentation instrument (Billerica, MA, USA) was used to determine the mechanical properties of the microzone at the interface of the composite material and the surrounding area. A Berkovich indenter was used, featuring a maximum load of 10 mN, loading time of 5 s, load-holding time of 2 s, and unloading time of 5 s. During the test, particles were randomly selected from the ground and polished surfaces. To characterize the mechanical properties and interface structure of the microzone near the interface of the composite material, the nanoindentation array was intersected with the interface at a small angle to maximize the number of indentations at the interface within the accuracy of the instrument.

The current general theoretical model for nanoindentation technology is that Oliver and Pharr [27] enhanced the technique of estimating the hardness and elastic modulus of materials by contact stiffness and depth based on the study of Doerner and Nix [28]. Through the analysis of the load-displacement curve obtained by the test, the contact stiffness (*S*) was able to be calculated by fitting the slope at the highest point of the unloading curve, and then the contact area (*A*) could be obtained. The hardness of the material was calculated from the maximum load (*P_max_*) and the contact area using the following equations.

(1) $$S={\left(\frac{dP}{dH}\right)}_{h=h_{max}}$$

(2) $$H=\frac{P_{max}}{A}$$

Reduced elastic modulus (*E_r_*) was calculated using Equation (3). The elastic modulus of most materials is much lower than that of diamond indenters, so *E_r_* can be utilized in place of elastic modulus (*E*).

(3) $$E_{r}=\frac{\sqrt{\pi }}{2\beta }\frac{S}{\sqrt{A}}$$

Quasi-static compression experiments at room temperature were performed using a universal material mechanical property tester (Zwick Z020, Ulm, Germany). The strain rate was 5 × 10^−4^/s. The compressed sample was cut into a cylinder with dimensions of Φ3 × 6 mm by wire electrical discharge machining. The two end faces of the compressed sample were parallel to each other.

## 3. Results and Discussion

### 3.1. Phase Analysis of Wp/Zr-BMGs with Different Wp Sizes

**Figure 1:**
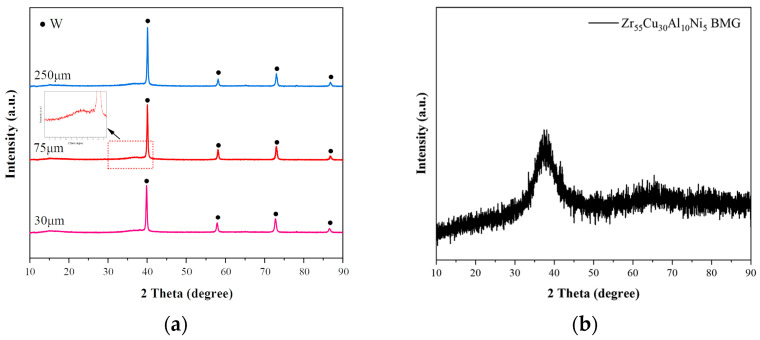
X-ray patterns of the composites with three Wp sizes: (**a**) Wp/Zr-BMGs; (**b**) Zr_55_Cu_30_Al_10_Ni_5_.

Figure 1 presents the X-ray patterns of the composites with three Wp sizes. As shown in the figure, the results for the composites with different Wp sizes are similar. Except for the crystallization peaks of tungsten particles and a diffuse scattering peak of the metallic glass (2θ = 38°), no other crystallization peaks are present, so the matrix is completely amorphous. This indicates that the Wp had no adverse effects on the formation of the bulk metallic glass during SPS.

### 3.2. Interface Structure of Wp/Zr-BMGs with Different Wp Sizes

**Figure 2:**
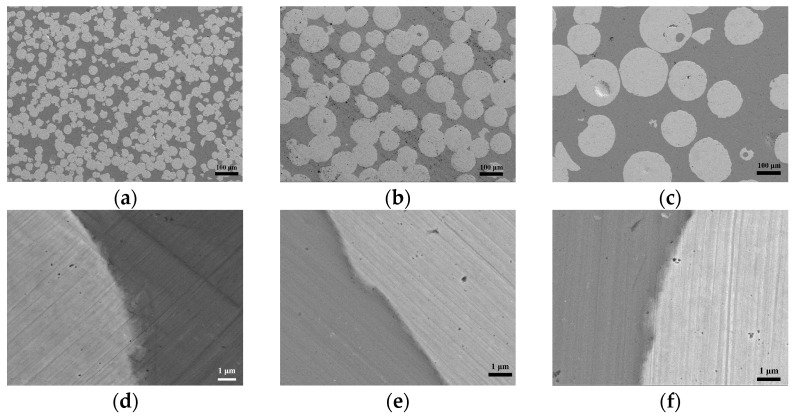
SEM images of the composites with three Wp sizes: (**a**,**d**) Wp—30 μm; (**b**,**e**) Wp—75 μm; (**c**,**f**) Wp—250 μm.

Figure 2 presents the SEM backscattered electron images of the three composites with different sizes of Wp. The circular light-grey area is Wp, and the dark-grey area is the Zr-BMG matrix. Notably, the distribution of Wp is relatively uniform, and the interface contact is good. The interfaces of the three different sizes of Wp and Zr-BMG are relatively complete, whereas certain positions are tortuous and staggered. The images reveal that no obvious intermetallic compounds precipitated during the preparation process, neither in the form of dispersed particles in the matrix nor as a continuous layer at the interface.

**Figure 3:**
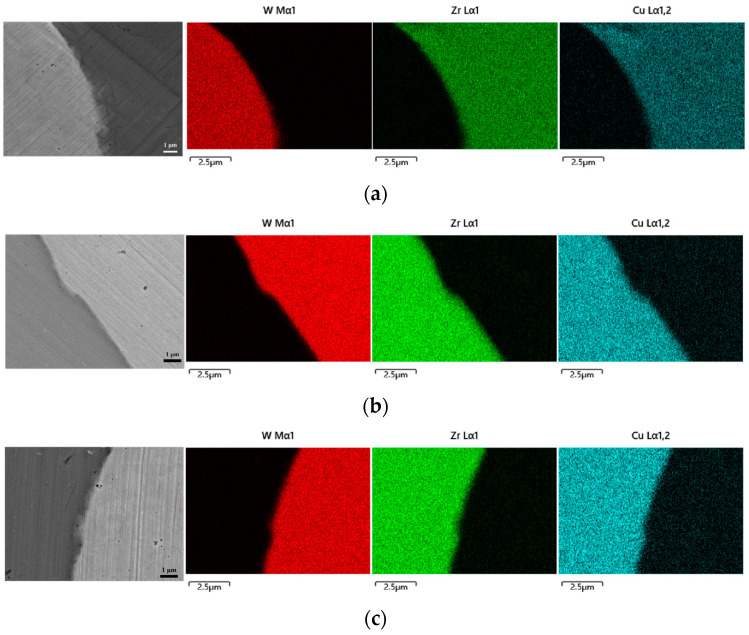
EDS maps of the composites with three Wp sizes near the interfaces: (**a**) Wp—30 μm; (**b**) Wp—75 μm; (**c**) Wp—250 μm.

**Figure 4:**
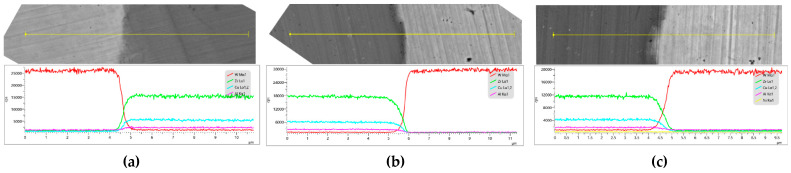
EDS line scans of the composites with three Wp sizes near the interfaces: (**a**) Wp—30 μm; (**b**) Wp—75 μm; (**c**) Wp—250 μm.

**Figure 5:**
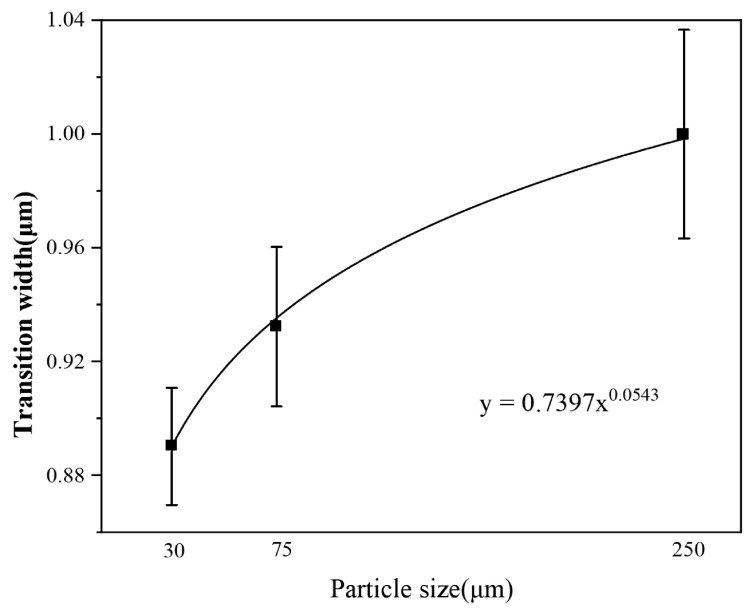
EDS line scan transition width of the composites with three Wp sizes.

**Figure 6:**
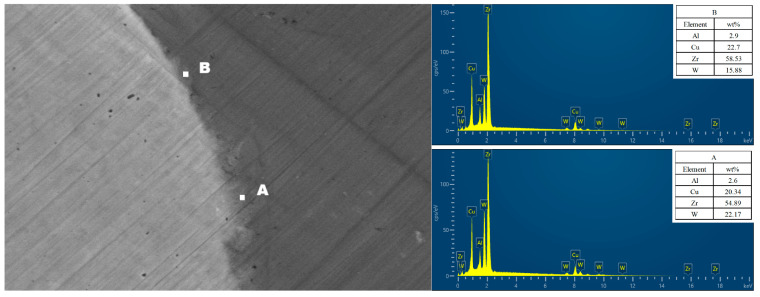
EDS spot scan of the composite with Wp size of 30 μm at the interface.

Figure 3 presents the elemental scanning maps obtained near the Wp/Zr-BMG interfaces, fabricated with different Wp sizes. The main elements at the interface of the three composites are clearly demarcated. Line scanning from Wp across the interface to the matrix, or vice versa, is depicted in Figure 4. The element content gradually transitions at the interface, displaying a uniform process without any plateaus. According to the abscissa axis, the average transition widths of element content of the three composites are approximately 0.891 μm (Wp: 30 μm), 0.933 μm (Wp: 75 μm), and 1.012 μm (Wp: 250 μm). Figure 5 demonstrates that the transition width increases as the Wp size increases, following an allometric equation. Based on these findings from Figure 1, Figure 2, Figure 3 and Figure 4, it can be concluded that no apparent intermetallic compounds grew and precipitated during the SPS process of Wp/Zr-BMGs with varying Wp sizes. It is regarded as a relatively ideal combination of two phases [29]. Figure 6 displays the EDS spectra obtained from spots A and B at the interface of the composite with a Wp size of 30 μm. Similar EDS spectra were obtained for spots within the interfaces of the composites, with the other two Wp sizes showing that there is both tungsten and elements of the BMG matrix such as Zr, Cu, and so on. The combination of Wp and Zr-BMG occurred through dissolution and diffusion. At the composite interface, there is both diffusion from the matrix elements to Wp and dissolution and diffusion from tungsten elements to the matrix. Following the theory of interface in metal composites, it can be inferred that the Wp/Zr-BMGs sintered by SPS exhibit a second type of interface, namely, a dissolution-diffusion interface [30]. Both the matrix and the reinforcement wetted the interface, and it involved mutual dissolution and diffusion to a certain degree. As wetting between the two phases is a primary condition for diffusion infiltration [31], under the same other conditions, the wetting contact angle decreases and wettability improves as the particle size increases [32], which enhances the diffusion infiltration between Wp and the BMG matrix. Consequently, the thickness of the interface diffusion layer increased with increasing Wp size.

### 3.3. Interfacial Mechanical Properties of Wp/Zr-BMGs with Different Wp Sizes

**Figure 7:**
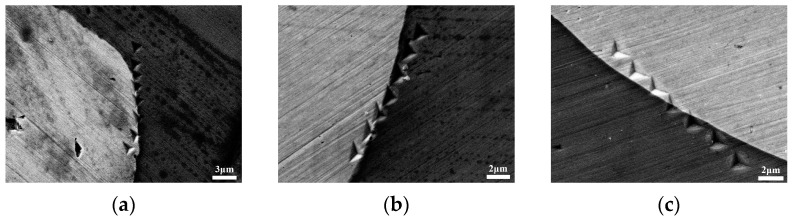
SEM images of the nanoindentation marks of Zr-BMGs with three Wp sizes: (**a**) Wp—30 μm; (**b**) Wp—75 μm; (**c**) Wp—250 μm.

Figure 7 displays the SEM images of the nanoindentation marks. The distance between the indentation center and the interfaces of the composites was measured using the image processing software Image J. Positive values represent distances from the indentation center on the matrix, while negative values indicate distances from the indentation center on Wp. The elastic modulus (E) and hardness (H) were measured at different positions near the interface.

**Figure 8:**
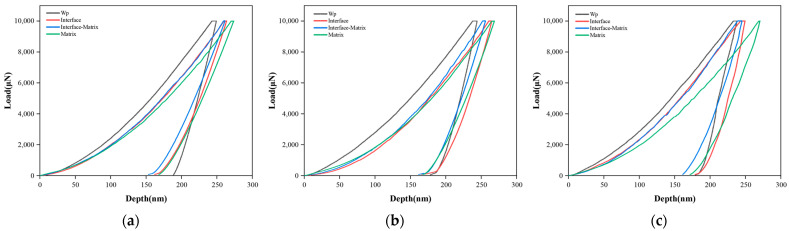
Load-displacement curves of Zr-BMGs with three Wp sizes: (**a**) Wp—30 μm; (**b**) Wp—75 μm; (**c**) Wp—250 μm.

As indicated in Figure 8, partial load-displacement curves of composites with three Wp sizes are produced. The indentations corresponding to the four curves were taken from Wp, the interface, the region of the matrix close to the interface, and the matrix. Taking the composites with a Wp of 30 μm as an example, the order of the maximum displacement of indentations at different positions from small to large is Wp < the matrix close to the interface < interface < matrix. The same trend applies to the samples with the other two Wp sizes. It can be seen that the maximum displacement of the indentation at the matrix close to the interface is less than that of the interface and the matrix; that is, the ability of the matrix close to the interface to resist external deformation is stronger than that of the interface and the matrix.

**Figure 9:**
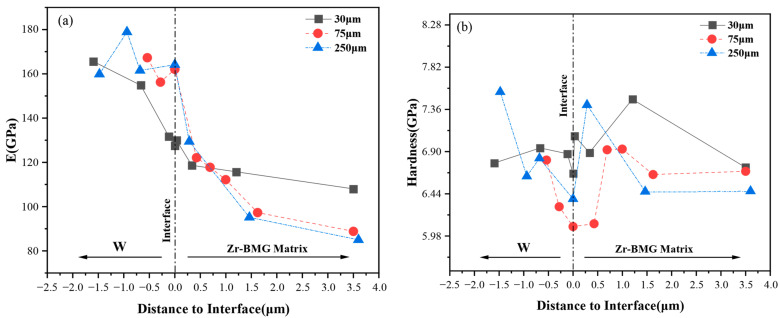
The mechanical properties of microarea at the interface of Zr-BMGs with three Wp sizes: (**a**) Elastic modulus; (**b**) Hardness.

Figure 9 illustrates the relationship between the elastic modulus (E) and hardness (H) near the interface of the Wp/Zr-BMGs with three Wp sizes and the distance from the indentation center to the interface. The origin (x = 0) on the transverse axis corresponds to the approximate position of the interface; the right side of the interface (x > 0) corresponds to the Zr-BMG matrix; and the left side of the interface (x < 0) corresponds to Wp. In the region x < 0 (Wp), the elastic modulus (E) of the samples with three Wp sizes generally formed a plateau at approximately 160 GPa, corresponding to the elastic modulus of Wp. At the interface and nearby matrix area, when 0 < x < ~1 μm, the elastic modulus values lie between those of the Wp and the matrix, where the distribution of values is relatively close. In the region of x > ~1 μm (matrix), the elastic modulus values of these samples also form a plateau, corresponding to the elastic modulus of the Zr-BMG matrix. In this region, the elastic modulus of the Wp size of 30 μm exhibits the highest value, followed by 75 μm and 250 μm. Regarding hardness, the hardness value decreases in the x < 0 (Wp) region with decreasing |x|, indicating that the hardness decreases as the indentation on Wp approaches the interface, with the hardness at the interface reaching the lowest value. In the region of 0 < x < ~1 μm, the hardness initially increases and then decreases away from the interface. In this region, the positions where the composites with different Wp values reach their maximum hardness values vary. The order of the width of the hardness growth area, from large to small, is 30 μm > 75 μm > 250 μm.

The addition of Wp enhances the elastic modulus and hardness of the matrix surrounding the interface compared with the Zr-BMG itself (Figure 9a,b, region 0 < x < ~1.3 μm compared with x > ~1.3 μm region). This phenomenon exhibits a certain size effect. Wp with a size of 30 μm demonstrates a relatively significant increase in the elastic modulus and hardness of the nearby matrix, whereas Wp with a size of 250 μm displays a relatively minimal increase in the elastic modulus and hardness of the nearby matrix. The range of this region is close to the thickness of the transition widths in Figure 4, so this region could be defined as the interfacial diffusion layer. The composite with a Wp size of 30 μm exhibits the most favorable interfacial diffusion layer among the three Wp sizes. In contrast, the composite with Wp measuring 250 μm may encounter issues due to an insufficiently close structure caused by the increased thickness of the diffusion layer or the increase of the free volume introduced into the Zr-BMG matrix. Compared to smaller-sized particles, the effect of improving the mechanical properties of the amorphous matrix diminishes with larger-sized particles. Therefore, under these specific sintering conditions, within a certain range of Wp sizes, the enhancement effect of Wp on the elastic modulus and hardness of the amorphous matrix exhibits a negative correlation with Wp size. Thus, smaller-sized Wp particles have a positive impact on the amorphous matrix.

### 3.4. Macromechanical Properties of Wp/Zr-BMGs with Different Wp Sizes

**Figure 10:**
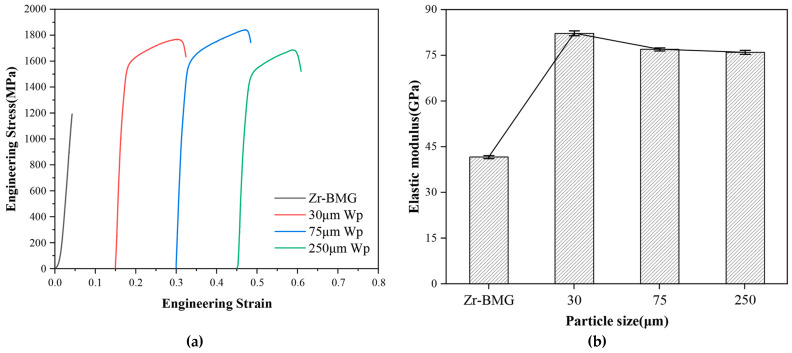
(**a**) Engineering stress-strain curves of Zr-BMG and Wp/Zr-BMGs with three Wp sizes; (**b**) Elastic modulus of Zr-BMG and Wp/Zr-BMGs with three Wp sizes.

Figure 10a presents engineering stress-strain curves of Zr-BMG and Wp/Zr-BMGs with three Wp sizes obtained from quasi-static room temperature compression tests. The fracture strength of Zr-BMG was 1192 MPa without plastic strain. For Wp/Zr-BMGs, the compressive strength and plasticity substantially increased. The elastic modulus can be estimated by fitting the elastic stage of each curve. The elastic modulus is the slope of the fitting line shown in Figure 10b. As observed in Figure 10b, the addition of Wp significantly improves the elastic modulus of Zr-BMG. Moreover, the order of the elastic modulus of composites with three Wp sizes is 30 μm > 75 μm > 250 μm. This difference is mainly due to the larger interfacial specific surface area of Wp/Zr-BMGs with smaller Wp sizes, which promotes a more effective transfer of stress into the reinforced phase.

## 4. Conclusions

1. The interface between Wp and Zr-BMG in Wp/Zr-BMGs with different Wp sizes exhibited a dissolution-diffusion interface without any apparent precipitated intermetallic compounds. This suggests that Wp particles have an ideal combination with Zr-BMGs after the SPS process.
2. The thickness of the diffusion layer at the interface increased as the Wp particle size increased. This positively correlated size effect can be attributed to the improved wettability between Wp and Zr-BMG. Larger Wp particles had better wetting contact and resulted in a thicker diffusion layer at the interface.
3. The incorporation of Wp particles not only enhanced the elastic modulus and hardness of the amorphous matrix near the interface but also improved macromechanical properties such as strength, plasticity, and elastic modulus. Notably, smaller-sized Wp particles demonstrated a more pronounced improvement in the mechanical properties of the Zr-BMG compared with larger-sized Wp particles.

These results suggest that Wp/Zr-BMGs with appropriate Wp particle sizes have the potential to enhance the interfacial properties and mechanical performance of the resulting material. The dissolution-diffusion interface between Wp and Zr-BMG contributes to the effective combination of the two phases, while the size-dependent effects highlight the importance of particle size optimization in achieving desired mechanical properties.

The study offered a basis for further research on tailoring the interfacial characteristics and optimizing the mechanical behavior of Wp/Zr-BMG systems. Future investigations can focus on exploring additional factors, such as processing parameters and different reinforcement materials, to further advance the understanding and applications of these composites in various engineering fields.

## Data Availability

The data presented in this study are available on request from the corresponding author. The data are not publicly available due to privacy.
